# Activation of Calcium/Calmodulin‐Dependent Kinase II in the Medial Prefrontal Cortex Mediates Spinal Cord Injury‐Related Cognitive and Affective Changes

**DOI:** 10.1002/brb3.71118

**Published:** 2025-12-10

**Authors:** Jian Qi, Chen Chen, Qian Gao, Sheng Sun

**Affiliations:** ^1^ Department of Orthopedics The 960th Hospital of PLA Jinan China; ^2^ Department of Pharmacy The Second Hospital of Shandong University Jinan China; ^3^ Jinzhou Medical University, The 960th Hospital of the Chinese People's Liberation Army Joint Logistic Support Force Postgraduate Training Base Jinan China; ^4^ Department of Orthopedics Beijing Chaoyang Hospital, Capital Medical University Beijing China

**Keywords:** anxiety‐like behavior, calcium/calmodulin‐dependent kinase II, cognitive impairments, depression‐like behavior, medial prefrontal cortex, rat, spinal cord injury

## Abstract

**Purpose:**

Research has documented a high prevalence of cognitive and affective impairments in individuals with spinal cord injury (SCI). However, the molecular mechanisms underlying these deficits remain poorly understood. In this study, to investigate the molecular basis of cognitive and affective dysfunctions following SCI, we examined the role of calcium/calmodulin‐dependent kinase II (CaMKII) activation, with a specific focus on its phosphorylated form (pCaMKII), using a rat model of SCI.

**Method:**

Experimental results demonstrated that SCI led to spatial memory deficits as well as depression‐ and anxiety‐like behaviors, as evidenced by performance in the Morris water maze (MWM), elevated plus maze (EPM), and forced swim test (FST). Compared to the sham group, increased levels of pCaMKII were observed in the medial prefrontal cortex (mPFC) at Day 56 after SCI. Moreover, inhibiting CaMKII phosphorylation via microinjection of KN‐93—a CaMKII activation inhibitor—into the mPFC alleviated depression‐like behavior and cognitive deficits, but not anxiety‐like behavior.

**Finds and Conclusion:**

These findings suggest that CaMKII activation in the mPFC may play an important role in mediating negative emotional states following SCI.

## Graphical abstract

Research has documented a high prevalence of cognitive and affective impairments in individuals with spinal cord injury (SCI). Our findings suggest that CaMKII activation in the mPFC might play an important role in mediating negative emotional states following SCI.

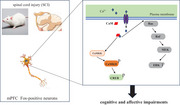

## 1 Introduction

Sensorimotor dysfunction and hyperpathia present considerable clinical challenges in the management of spinal cord injury (SCI) (Sanganahalli et al. 2021). Moreover, a substantial body of evidence indicates clinically significant affective and cognitive impairments are highly prevalent following SCI (Craig et al. 2009; Mokhtari and Uludag 2024; Post and van Leeuwen 2012; Wu et al. 2014), with an estimated 40% of individuals at risk for developing these disorders (Zhang et al. 2025). Although previous research on SCI has primarily focused on afferent and efferent pathway changes, accumulating evidence reveals significant supratentorial alterations in brain regions such as the primary motor cortex, prefrontal cortex (PFC), and anterior cingulate cortex (Cao et al. 2026). Converging evidence establishes the PFC as a critical hub for orchestrating emotion, affect, and cognition in the adult brain (Maviel et al. 2004; Ren et al. 2025). The medial prefrontal cortex (mPFC), a well‐established contributor to depression and cognitive deficits in neuropsychiatric disorders (Li et al. 2025), is similarly impaired by SCI, leading to analogous disruptions in emotional and cognitive processing (Kazemi et al. 2025).

Accumulating evidence demonstrates that cognitive and emotional deficits following SCI are associated with supraspinal synaptic reorganization, driven by injury‐induced alterations in the molecular mechanisms underlying synaptic plasticity (Sanchez‐Ventura et al. 2022; Yin et al. 2023). Emerging evidence indicates that synaptic deficits observed in PFC subregions reflect impaired long‐term potentiation (LTP), particularly manifest as reduced plasticity at excitatory pyramidal synapses (Algaidi 2025; Marsden 2013). Furthermore, stress and negative emotional states may disrupt synaptic plasticity through modifications in multiple signal transduction pathways (Marsden 2013). Therefore, we have initiated research to determine whether SCI influences molecular systems that modulate neuronal plasticity in the mPFC. Understanding this mechanism may improve the comprehension of cognitive deficits and depression post‐SCI and provide a foundation for therapies aimed at enhancing functional recovery.

Calcium/Calmodulin‐dependent protein kinase II (CaMKII) is a critical signaling molecule essential for LTP (Yasuda et al. 2022). CaMKII is initially activated by glutamate receptors and subsequently phosphorylated via IP3‐mediated pathways. This phosphorylation enables CaMKII to modulate synaptic function through the phosphorylation of membrane receptors. CaMKII acts as a key upstream regulator of MAP kinase signaling pathways—such as p38 MAPK, ERK1/2, and CREB—thereby influencing downstream gene expression and synaptic plasticity (Jiang et al. 2021). Furthermore, substantial evidence indicates that under conditions of SCI—which induces extensive neuroinflammation in the brain linked to cognitive and affective alterations—multiple kinase pathways, including CaMKII, become activated (Wu et al. 2014). Previous research has shown that CaMKII phosphorylation activates downstream pathways that contribute to the pathological processes following SCI (Gwak et al. 2013).

Although SCI is known to cause cognitive and emotional deficits, the potential role of CaMKII activation within the mPFC in these impairments remains unexplored. To address this gap, we examined the impact of SCI on cognitive and affective behaviors in rats and assessed the contribution of CaMKII activation to these outcomes. Furthermore, by microinjecting KN‐93—a highly selective CaMKII inhibitor—into the mPFC, we investigated whether CaMKII signaling might play a specific role in mediating SCI‐induced abnormal emotional responses.

## 2 Materials and Methods

### 2.1 Animals and Models

All experimental procedures were performed on male Sprague–Dawley rats weighing 200–250 g. The study was conducted in accordance with the Guidelines for the Care and Use of Mammals in Neuroscience and Behavioral Research and was approved by the Animal Ethics Committee of the Military 960th Hospital (Ethical Approval No.: 200018). The animals were housed individually under controlled conditions at 22–24°C with a 12‐h light/dark cycle.

To establish the SCI model, rats were anesthetized using sodium pentobarbital (45 mg/kg, i.p.). A laminectomy was performed at the T7–T9 vertebral level to expose the spinal cord. The cord was elevated with a curved probe and completely transected at the T8–T9 junction. The surgical site was irrigated thoroughly, and the wound was closed in layers with sutures.

### 2.2 Experimental Design and Microinjections

**FIGURE 1:**
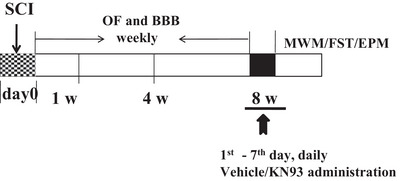
Basic experimental design schematic diagram. The behavioral tests and the duration time for KN93 administration are depicted. BBB, Basso, Beattie, and Bresnahan; EPM, elevated plus maze; FST, forced swimming test; MWM, Morris water maze; OF, open field; SCI, spinal cord injury; W, week.

The experimental design and timeline are summarized in Figure 1. A total of 104 rats were used and assigned to groups according to a randomized block design: (1) Sham‐operated control group; (2)–(10) SCI groups assessed at different time points: Days 1, 7, 14, 21, 28, 35, 42, 49, and 56 post‐SCI; (11) vehicle‐treated SCI group at Week 8: received daily vehicle administration on consecutive 7 days; (12) KN‐93‐treated SCI group at Week 8: received daily KN‐93 administration on consecutive 7 days; (13) KN‐93‐treated sham group: received daily KN‐93 administration on consecutive 7 days.

For microinjection, on the first day at Week 8, the rats were anesthetized via intraperitoneal injection of sodium pentobarbital (50 mg/kg) dissolved in 0.9% (w/v) saline and placed in a supine position in a stereotaxic apparatus (Kopf Instruments, Tujunga, CA, USA). The head was secured using ear and bite bars, and an incision was made to expose the skull. Stainless‐steel 22‐ga guide cannulae (PlasticsOne, Roanoke, VA, USA) were bilaterally implanted into mPFC. The stereotaxic coordinates were as follows: mPFC: +2.9 mm AP, ±1.9 mm ML, −2.8 mm DV. The cannulae were fixed to the skull with four screws and dental cement. A dummy cannula was inserted into each guide cannula to maintain patency. After the procedure, the rats regained consciousness and were allowed to recover in their home cages before behavioral testing began. KN‐93 (5 µg/10 µL, dissolved in 5% DMSO; Abcam, Cambridge, MA, USA) or vehicle was administered daily for 1 min continuing for 7 days. All behavioral assessments were conducted under double‐blind conditions. The overall experimental timeline is illustrated in Figure 1.

### 2.3 The Evaluation of Motor Function

Rats were evaluated for hindlimb functional recovery beginning on Day 1 after SCI and subsequently assessed on a weekly basis for 8 weeks using the Basso, Beattie, and Bresnahan (BBB) locomotor rating scale and open field (OF) testing (Basso et al. 1995). The BBB locomotor scale, which ranges from 0 (complete paralysis) to 21 (normal locomotion), was used to assess functional recovery. Average scores were calculated to represent the progression of locomotor recovery following SCI. In addition, spontaneous locomotor activity was evaluated weekly for 8 weeks post‐injury using an OF test. Each rat was placed individually in a corner of a square chamber (23 × 23 cm^2^), facing the wall, and allowed to explore freely for 5 min. The total distance traveled and average speed were recorded via an overhead video tracking system.

### 2.4 Behavioral Tests

PFC‐dependent spatial learning and memory were assessed using the Morris water maze (MWM), a widely established behavioral paradigm for evaluating cognitive processes and their underlying neural mechanisms (Qi et al. 2014). The MWM test was initiated at 8 weeks post‐SCI. A circular pool, situated in a temperature‐controlled room, was filled with opaque water maintained at 20–23°C. The pool was divided into four quadrants and surrounded by prominent extra‐maze visual cues. A hidden cylindrical platform was positioned in the target quadrant (southeast), submerged 2 cm below the water surface. Twenty‐four hours prior to formal testing, rats were habituated to the pool environment. During the spatial acquisition phase, animals used extra‐maze cues to locate the hidden platform across 16 trials administered over four consecutive days (four trials per day, with a 20‐s intertrial interval). Each trial allowed a maximum of 60 s to find the platform; successful rats remained on it for 30 s, whereas those failing were guided onto it and allowed to remain for 20 s. Escape latency was recorded as the primary measure of learning performance. Learning trials were conducted daily on Days 1–7 of Week 8. To assess spatial memory, the platform was removed for the probe trial, during which the percentage of time spent in the target quadrant was recorded. All swim paths were tracked using a digital video camera mounted directly above the pool.

The Forced Swim Test (FST) was employed to assess depression‐like behavior (Y. W. Liu et al. 2015). Rats were placed in a transparent cylindrical tank (30 cm in height, 10 cm in diameter) filled with water to a depth of 20 cm and allowed to swim for 5 min. Following the test, the animals were gently dried with tissue and returned to their home cages. All sessions were recorded with a video camera. The total immobility time during the 5‐min test period was quantified. The FST was conducted during the eighth week post‐SCI.

The elevated plus maze (EPM), consisting of two open arms and two enclosed arms (each 45 × 10 cm^2^, with 66 cm high walls for the closed arms), was used to assess anxiety‐like behavior in rats. Each rat was initially placed in the central platform (10 × 10 cm^2^) facing a closed arm. Behavior was recorded for 5 min using an overhead video camera. The total distance traveled and time spent in the open and closed arms were quantified as measures of anxiety. Anxiety‐like behavior was evaluated on the basis of the percentage of time spent in the open arms relative to the total time in all arms, as well as the percentage of open arm entries relative to the total number of entries.

### 2.5 Immunohistochemical Staining of Activated Calcium/Calmodulin‐Dependent Kinase II (pCaMKII) and Fos

Rats were transcardially perfused with isotonic saline followed by 4% paraformaldehyde in 0.1 M phosphate buffer (PB, pH 7.4). Brains were then removed, post‐fixed in the same fixative for 4 h, and cryoprotected in 30% sucrose for 48 h at 4°C. Coronal sections (30‐µm thick) were prepared from the forebrain using a freezing microtome (Kryostat 1720; Leitz, Mannheim, Germany). Double immunohistochemical staining was performed to detect pCaMKII and Fos. Briefly, sections were incubated in 10% (v/v) fetal bovine serum (FBS; F6178, Sigma, St. Louis, MO, USA) for 1 h. They were then incubated sequentially in the following antibody mixtures: (1) a combination of mouse anti‐Fos (1:500, AB11959; Abcam) and rabbit anti‐pCaMKII (1:200, AB32678; Abcam, Cambridge, MA, USA) at 4°C for 24 h; (2) a mixture of goat anti‐mouse IgG conjugated to Alexa Fluor 594 (1:400; Molecular Probes, Oregon) and goat anti‐rabbit IgG conjugated to Alexa Fluor 488 (1:400; Molecular Probes) in PBS containing 0.3% Triton X‐100. After three washes in PBS, sections were mounted onto gelatin‐coated slides and coverslipped with a mounting medium consisting of 0.1 M PBS, 5% (v/v) glycerin, and 2.5% (w/v) triethylenediamine. Imaging was performed using a confocal laser‐scanning microscope (Fluoview 1000, Olympus, Tokyo, Japan), and digital images were acquired with FLUOVIEW software (Olympus).

### 2.6 Western Immunoblotting

To examine changes in pCaMKII expression following SCI using Western blot analysis, rats were anesthetized with sodium pentobarbital (60 mg/kg) and euthanized by decapitation. Protein extracts (50 µg) from the mPFC were separated by electrophoresis and transferred onto PVDF membranes. The membranes were probed with the following primary antibodies: anti‐pCaMKII (rabbit, 1:1000; Abcam), anti‐total CaMKII (tCaMKII, mouse, 1:1000; AB22609, Abcam), anti‐pERK1/2 (rabbit, 1:1000; 12185, CST), anti‐total ERK1/2 (tERK, rabbit, 1:1000; 4695, CST), anti‐Fos (mouse, 1:1000; Abcam), and anti‐β‐actin (mouse, 1:1000; Sigma). Subsequently, membranes were incubated with horseradish peroxidase‐conjugated secondary antibodies, including anti‐rabbit (1:3000) and anti‐mouse (1:5000). Protein signals were detected using an enhanced chemiluminescence (ECL) system (Amersham) and quantified with ImageJ software.

### 2.7 Statistical Analysis

Statistical analyses were performed using the following methods: Data from the elevated EPM, the number of pCaMKII‐immunoreactive neurons, and protein levels of pCaMKII, pERK, and Fos were analyzed by one‐way ANOVA. The BBB scores, spatial memory performance, and OF indices were evaluated using two‐way ANOVA. The FST and learning performance data were analyzed with three‐way ANOVA. Post hoc comparisons between groups were conducted using the Student–Newman–Keuls method. All tests were two‐sided, and a *p* value of less than 0.05 was considered statistically significant.

## 3 Results

### 3.1 Motor Function Is Deficit After SCI

**FIGURE 2:**
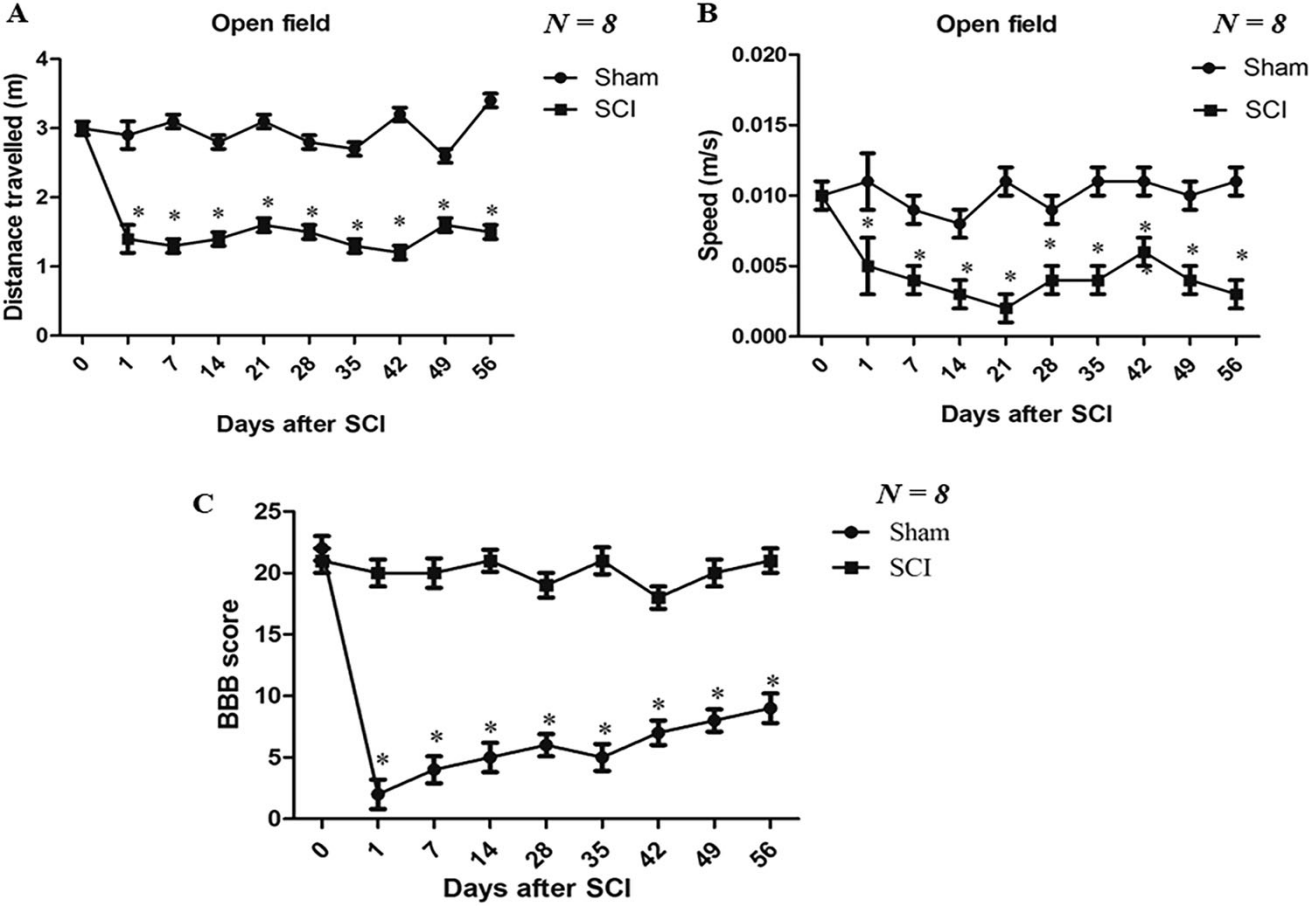
The OF test showed spontaneous locomotor movement and hindlimb functions. (A) and (B) traveled distance and speed. SCI rats resulted in a significant reduced distance traveled and walking speed compared with sham rat (*p < 0.05, compared to the sham). (C) Hindlimb locomotor function was evaluated using the BBB score. SCI rats resulted in a significant increased BBB score compared with sham rat (*p < 0.05, compared to the sham). *N* = 8 for each group. BBB, Basso, Beattie, and Bresnahan; SCI, spinal cord injury.

Spontaneous locomotor activity was assessed using the OF test following SCI. Compared to sham‐operated rats, SCI rats exhibited a significant reduction in both total distance traveled and average walking speed during the 5‐min test period (*p* < 0.05; Figure 2A,B). Hindlimb motor function was evaluated on Day 1 post‐SCI and weekly for 8 weeks using the BBB locomotor rating scale (Figure 2C). Statistical analysis revealed significantly lower BBB scores in SCI groups relative to the sham group (*p* < 0.05).

### 3.2 SCI Rats Had Significantly Greater Expression of pCaMKII in the mPFC in Fos‐Positive Neurons

**FIGURE 3:**
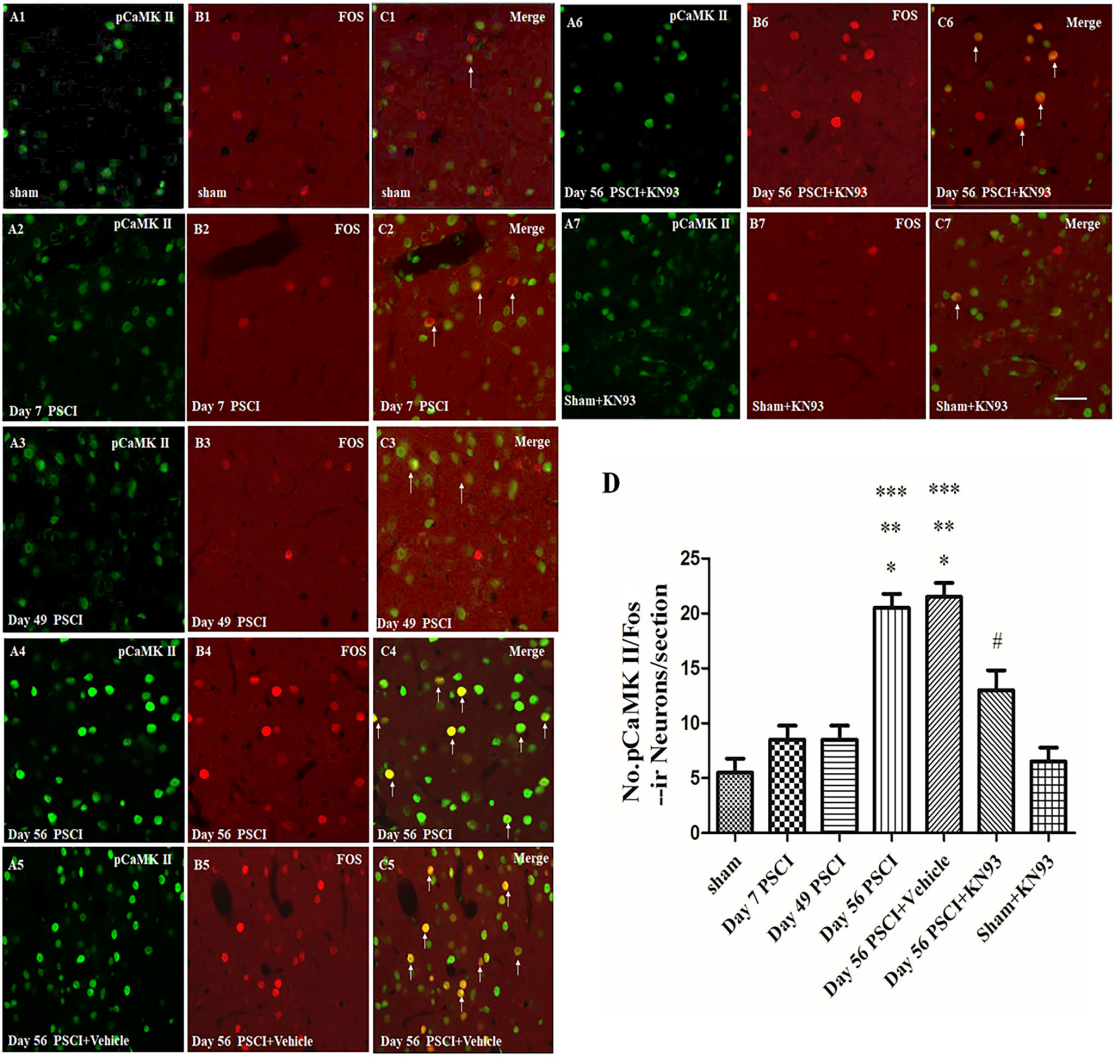
Typical microphotographs showing the expression of phosphorylated CaMKII (pCaMKII) and Fos in the mPFC. Scale bars = 100 µm (A1–C1, A2–C2, A3–C3, A4–C4, A5–C5, A6–C6, A7–C7). (D) Comparison of the number of pCaMKII‐positive neurons in Fos‐positive neurons between different groups. Data are means ± SEM of the number of neurons per section in the mPFC (*p < 0.05, compared to the sham group; **p < 0.05, compared to Day 7 PSCI group; ***p < 0.05, compared to Day 49 PSCI group; #*p* < 0.05, compared to Day 56 PSCI group). Single arrows show pCaMKII/Fos‐positive neurons.

To investigate whether CaMKII activation is associated with cognitive decline and depression‐/anxiety‐like behaviors after SCI, we compared the expression of pCaMKII in neurons of the mPFC at various post‐SCI time points using immunohistochemical staining. Immunostaining for pCaMKII showed significant differences in the number of pCaMKII‐positive neuronal bodies in the mPFC across groups (*F*_(6, 24)_ = 100.7, *p* < 0.001; Figure 3). Morphological analysis of pCaMKII expression within Fos‐positive neurons—where Fos protein serves as a marker of neuronal activation—revealed that SCI rats exhibited significantly higher pCaMKII levels in Fos‐positive mPFC neurons at Day 56 post‐SCI compared to sham‐operated rats (*p* < 0.05), Day 7 post‐SCI (*p* < 0.05), and Day 49 post‐SCI (*p* < 0.05). In contrast, rats treated with KN‐93 at Day 56 post‐SCI showed significantly fewer pCaMKII‐activated neurons compared to the untreated Day 56 SCI group (*p* < 0.05).

### 3.3 More Activated CaMKII Protein in the mPFC Corresponds to Cognitive Decline and Depression/Anxiety‐Like Behavior Following SCI

**FIGURE 4 (:**
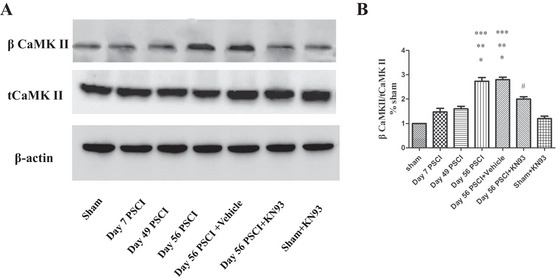
A) Immunoblots of pCaMKII/CaMKII in each group in the mPFC. (B) Densitometry analysis of bands relating to pCaMKII/CaMKII. Compared to the sham group, pCaMKII/CaMKII was upregulated at the protein level following Day 56 PSCI. Furthermore, pCaMKII/CaMKII was downregulated on Day 56 PSCI+KN93 compared to Day 56 PSCI‐treated rat (*p < 0.05, compared to the sham group; **p < 0.05, compared to Day 7 PSCI group; ***p < 0.05, compared to Day 49 PSCI group; #*p* < 0.05, compared to Day 56 PSCI group).

To elucidate the molecular mechanisms underlying SCI‐induced cognitive impairment and depression‐/anxiety‐like behaviors, we evaluated the effect of SCI on CaMKII activation within the mPFC. Tissue samples from the mPFC were collected from sham and SCI rats at various time points post‐injury. Western blot analysis revealed significant differences in the ratio of pCaMKII to total CaMKII among the groups (*F*_(6, 21)_ = 74.78, *p* < 0.001). Specifically, SCI rats exhibited a significantly higher pCaMKII/CaMKII ratio at Day 56 post‐SCI compared to the sham group (*p* < 0.05), Day 1 post‐SCI (*p* < 0.05), and Day 49 post‐SCI (*p* < 0.05) (Figure 4A,B). In contrast, no significant differences were observed in non‐phosphorylated CaMKII expression across groups (*p* > 0.05), indicating that SCI enhances CaMKII phosphorylation rather than its overall expression. Administration of KN‐93 at Day 56 post‐SCI markedly reduced pCaMKII activation compared to the untreated Day 56 SCI group (*p* < 0.05), whereas KN‐93 treatment in sham‐operated animals did not significantly alter CaMKII phosphorylation levels relative to the sham group (*p* > 0.05).

### 3.4 Inhibition of CaMKII With KN‐93 Attenuated Cognitive Decline Following SCI

**FIGURE 5 (:**
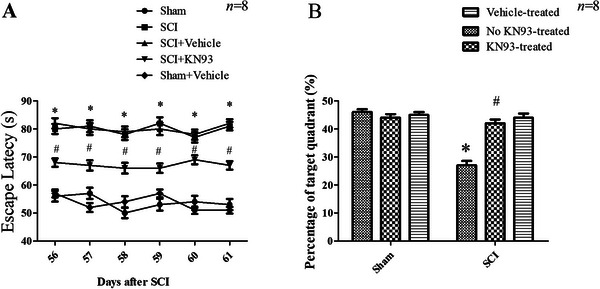
A) Comparison of SCI and KN93 treatment on mean escape latency. The MWM test showed the differences of the mean escape latency (*p < 0.05, compared to the sham group; #*p* < 0.05, compared to the SCI group). (B) The MWM test showed the comparison of the percentage of time spent in the target quadrant in different groups (*p < 0.05, compared to the sham; #*p* < 0.05, compared to the no KN93‐treated group). *N* = 8 for each group. SCI, spinal cord injury.

To evaluate whether SCI induces cognitive decline, we performed the MWM test. As shown in Figure 5A, escape latency was significantly influenced by group (*F*_(4, 36)_ = 12.27, *p* < 0.001), test day (*F*_(4, 36)_ = 127.34, *p* < 0.001), and treatment (*F*_(4, 36)_ = 576, *p* < 0.001). Rats in Day 56 post‐SCI group exhibited significantly longer escape latencies compared to the sham group (*p* < 0.05). Microinjection of KN‐93 into the mPFC of Day 56 post‐SCI rats for six consecutive days significantly shortened the latency to locate the hidden platform relative to untreated SCI rats (*p* < 0.05). On the fifth training day, spatial memory was assessed using a probe trial. Both group (*F*_(5, 44)_ = 107.8, *p* < 0.001) and treatment (*F*_(5, 44)_ = 218.6, *p* < 0.001) had a significant effect on the time spent in the target quadrant (Figure 5B). SCI rats at 8 weeks spent less time in the target quadrant than sham rats (*p* < 0.05). Chronic KN‐93 treatment reversed this deficit, as treated SCI rats spent significantly more time in the target quadrant compared to untreated SCI controls (*p* < 0.05).

### 3.5 Inhibition of CaMKII With KN‐93 Attenuated Depression‐Like but Not Anxiety‐Like Behavior Following SCI

**FIGURE 6:**
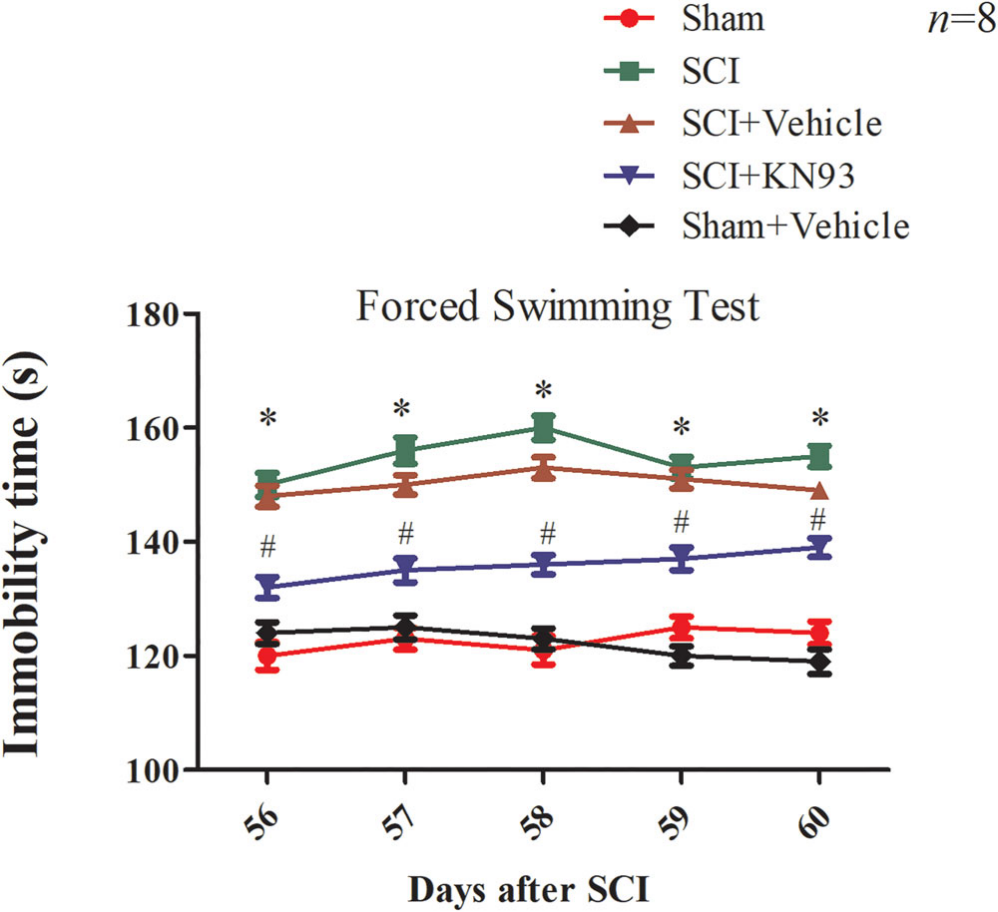
FST shows that KN93 administration relieves depression‐like behavior in SCI rat. The immobile time is increased in Days 56–59 PSCI, and KN93 administration reduces the immobile time to the level similar to that of sham rat. Vehicle administration has no effect on the immobile time of SCI rat (*p < 0.05, compared to the sham; #*p* < 0.05, compared to the SCI‐treated group). *N* = 8 for each group. *N* = 8 for each group. SCI, spinal cord injury.

Our results demonstrate that SCI induces long‐term depression‐like behavior, as measured by the FST. Sham‐operated rats exhibited an immobility time of approximately 80 s. In contrast, rats in Day 56 post‐SCI group showed a significant increase in immobility time compared to sham controls (*p* < 0.05). However, chronic administration of KN‐93 into the mPFC over 6 days significantly reduced immobility time in Day 56 post‐SCI rats relative to untreated SCI animals (*p* < 0.05; Figure 6).

**FIGURE 7:**
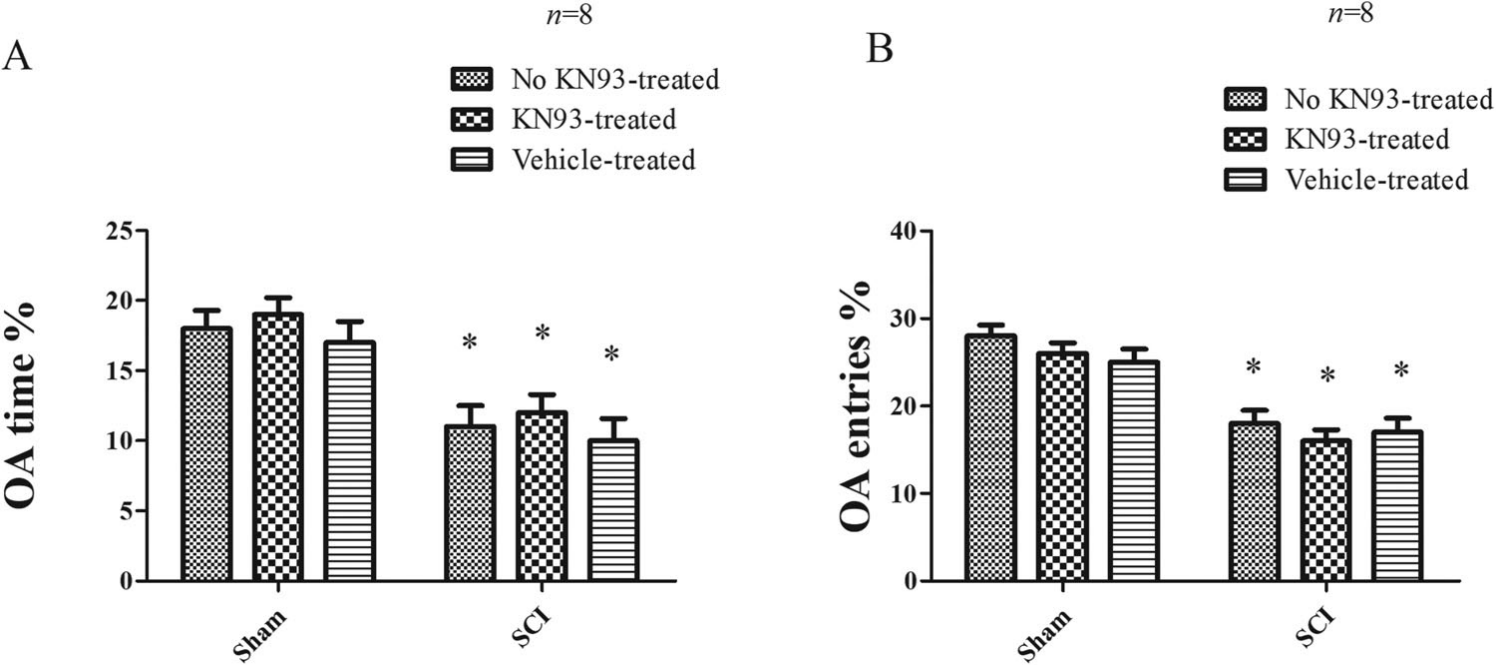
(A) The EPM test showed the comparison of open‐arm (OA) time/total time among different groups (*p < 0.05, compared to the no KN93‐treated sham group; #*p* < 0.05, compared to the vehicle‐treated SCI group). (B) The EPM test showed the comparison of open‐arm (OA) entries/total entries in different groups (*p < 0.05, compared to the sham group). *N* = 8 for each group. SCI, spinal cord injury.

In the EPM, one‐way ANOVA revealed significant differences in the percentage of time spent in the open arms (OA time%; *F*_(5, 44)_ = 98.2, *p* < 0.001) and the percentage of open arm entries (OA entries%; *F*_(5, 44)_ = 135.2, *p* < 0.001). Both OA time% and OA entries% were significantly lower in Day 56 post‐SCI rats compared to the sham group (*p* < 0.05). Notably, 6‐day chronic KN‐93 treatment in the mPFC did not reverse these reductions in open arm exploration (*p* < 0.05; Figure 7).

### 3.6 More Activated ERK, CREB Protein Levels in the mPFC Correspond to Cognitive Decline and Depression/Anxiety‐Like Behavior Following SCI

**FIGURE 8:**
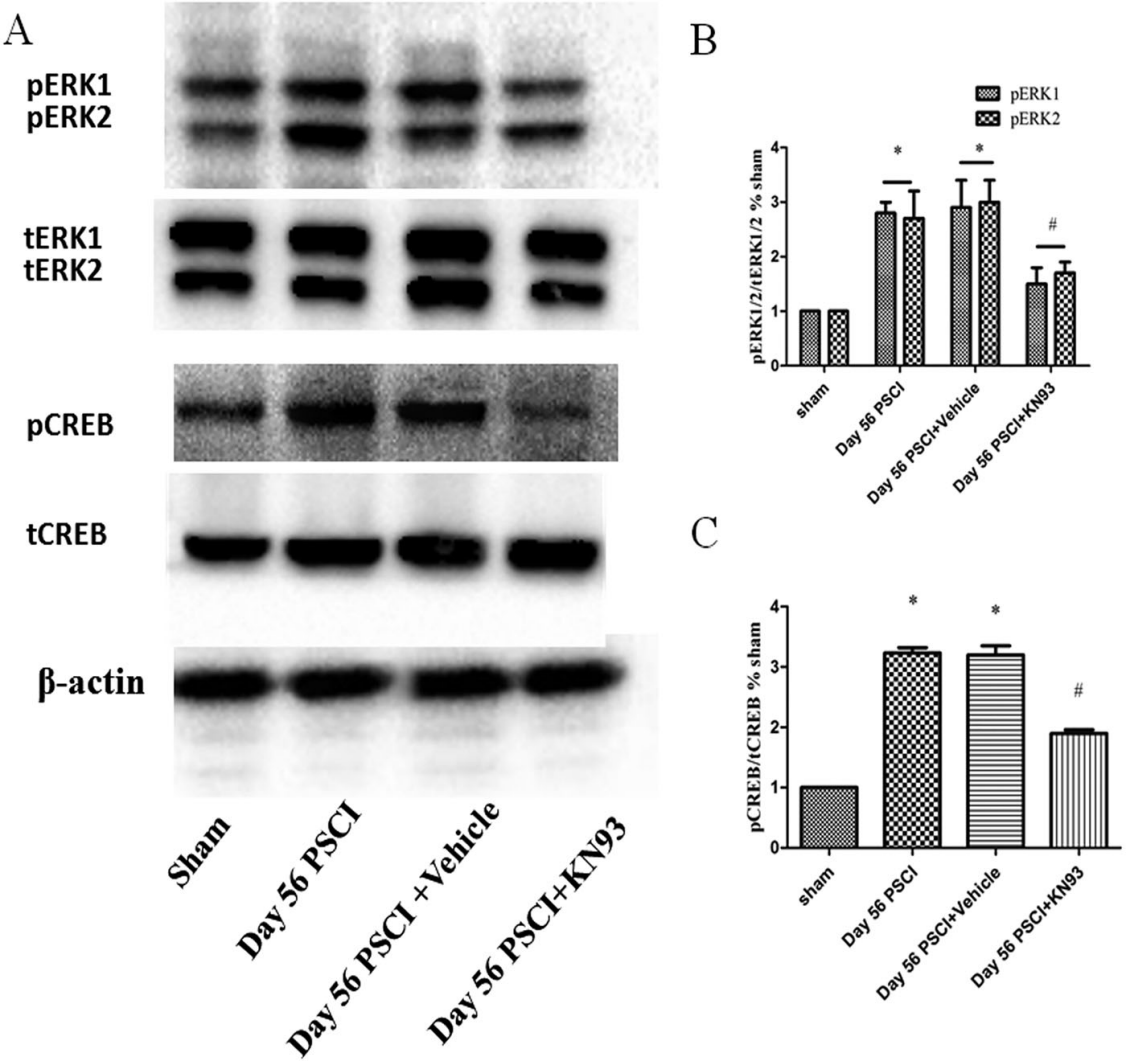
(A) Immunoblots of pERK1/2/tERK1/2, pCREB/tCREB in the mPFC. (B) The analysis of bands about pERK1/2/tERK1/2. Compared to the sham group, the protein level of pERK1/2/tERK1/2 was upregulated following Day 56 PSCI. Furthermore, pERK1/2/tERK1/2 was downregulated on Day 56 PSCI + KN93 compared to Day 56 PSCI‐treated rats. (C) Densitometry analysis of bands corresponding to pCREB/tCREB. Compared to the sham group, pCREB/tCREB was upregulated at the protein level following Day 56 PSCI. Furthermore, pCREB/tCREB was downregulated on Day 56 PSCI + KN93 compared to Day 56 PSCI‐treated rats (*p < 0.05, compared to the sham group; #*p* < 0.05, compared to Day 56 PSCI group).

To investigate downstream signaling pathways associated with CaMKII activation, we measured the protein levels of pERK1/2/tERK1/2 and pCREB/tCREB in the mPFC. One‐way ANOVA revealed significant differences among groups in the ratios of pCREB/tCREB and pERK1/2/tERK1/2 (*F*_(3, 12)_ = 67.75, *p* < 0.001). Rats in Day 56 post‐SCI group showed significantly elevated pERK1/2/tERK1/2 and pCREB/tCREB levels compared to sham‐operated rats (*p* < 0.05; Figure 8A,B). In contrast, no significant differences were observed in non‐phosphorylated ERK1/2 or CREB expression across groups (*p* > 0.05), indicating that SCI enhances the phosphorylation, but not the total expression, of these signaling molecules. Both pERK1/2 and pCREB activation were markedly reduced in Day 56 post‐SCI group treated with KN‐93 compared to the untreated Day 56 SCI group (*p* < 0.05). Furthermore, CaMKII phosphorylation levels in the KN‐93‐treated SCI group were significantly lower than those in the vehicle‐treated SCI controls (*p* < 0.05; Figure 8A,B).

## 4 Discussion

Employing a rat model of SCI, the present study yielded the following findings: (1) SCI animals exhibited long‐term locomotor deficits, supported by OF test results showing significantly reduced travel distance and walking speed, as well as decreased BBB scores compared to the sham group. These observations are consistent with previous reports in the literature (Wu et al. 2014); (2) in the MWM, SCI animals displayed significantly prolonged escape latency and reduced time spent in the target quadrant, indicating the presence of spatial learning and memory deficits; (3) the FST revealed a significant increase in immobility time among SCI rats, demonstrating depression‐like behavior following SCI; (4) EPM testing showed a reduction in both open arm time percentage and open arm entries in SCI rats, consistent with anxiety‐like behavior; (5) immunoblot and immunohistochemical analyses indicated significant upregulation of pCaMKII in the mPFC at 56 days post‐SCI, which was correlated with cognitive decline and emotional disturbances; (6) chronic administration of the CaMKII inhibitor KN‐93 into the mPFC suppressed CaMKII phosphorylation, ameliorated cognitive deficits and depression‐like behavior, but did not significantly improve anxiety‐like behavior. In addition, phosphorylation of ERK and CREB was also elevated in the mPFC at 56 days post‐SCI.

Emerging research is beginning to elucidate how SCI influences molecular mechanisms underlying synaptic plasticity in the brain (Sanchez‐Ventura et al. 2022). SCI induces plasticity across the central nervous system, including remote supratentorial regions. Accumulating evidence has revealed localized alterations in brain areas linked to SCI‐induced hyperesthesia, involving chronic inflammatory responses associated with neural plasticity and electrophysiological changes (Wu et al. 2016). SCI is frequently accompanied by psychological morbidity, manifesting as cognitive decline and depression‐ or anxiety‐like behaviors (Wang et al. 2025). Although substantial evidence indicates that SCI can lead to cognitive decline and depression‐ or anxiety‐like behaviors, the underlying molecular mechanisms remain poorly understood. Previous molecular and cellular studies have implicated alterations in multiple signaling pathways, neuroplasticity, neurometabolic activity, and neuroinflammatory responses. In this study, we therefore sought to investigate potential mechanisms using an SCI model. A growing body of research has shown that region‐specific changes in synaptic structure and function contribute to cognitive and emotional deficits (Marsden 2013). Numerous studies suggest that alterations in synaptic plasticity and function represent a key neurological mechanism underlying cognitive impairment in animal models of aging, injury, and disease (Zhao et al. 2025). Depression is a complex and heterogeneous disorder whose neurobiology involves coordinated changes across multiple CNS regions, including the mPFC, ACC, thalamus, hippocampus, and amygdala. (Marsden 2013).

The mPFC plays a critical role in mediating the effects of stress on cognitive function and psychopathology. Moreover, impaired mPFC function and plasticity represent a core pathological feature of numerous neuropsychiatric disorders (Popoli et al. 2012). In the CNS, synaptic efficacy is modulated by NMDA receptors, whose activity is regulated through phosphorylation by protein kinases. Furthermore, calcium dysregulation has long been implicated in cognitive dysfunction (Yang et al. 2025). Elevated intracellular Ca^2+^ concentrations, resulting from influx through activated NMDA receptors, lead to the phosphorylation and activation of CaMKII—a key signaling kinase (Nicoll and Schulman 2023). Previous studies have demonstrated that once phosphorylated, CaMKII maintains its activity via autophosphorylation, enabling sustained signaling even after the initial calcium stimulus has subsided (Zeitz et al. 2004) and even after Ca^2+^ concentrations return to baseline. Substantial evidence indicates that under conditions of SCI—which induces extensive neuroinflammation in the brain linked to cognitive and affective alterations—multiple kinase pathways, including CaMKII, become activated. CaMKII stimulation can lead to the activation of MAPKs, such as ERK1/2 and p38, subsequently inducing phosphorylation of transcription factors and alterations in gene expression (Ding et al. 2022; T. Liu et al. 2024). As a member of the MAPK family, ERK1/2 is highly sensitive to stress and plays a crucial role in cognitive function and mood regulation (Zheng et al. 2008), and our previous studies indicated that ERK1/2 activation is involved in SPS‐induced cognitive impairment (Qi et al. 2014). In the present study, behavioral analyses revealed that rats with SCI displayed cognitive deficits as well as depression‐ and anxiety‐like behaviors. These negative emotional behaviors and cognitive impairments were associated with elevated CaMKII activation in the mPFC. These findings are consistent with previous studies indicating that SCI leads to cognitive and affective dysfunction. Attenuation of CaMKII phosphorylation via intra‐mPFC infusion of KN‐93 ameliorated depression‐like behavior and cognitive deficits, but not anxiety‐like behavior. Nevertheless, we cannot rule out the possibility that other kinases, also modulated by the pharmacological treatment, contribute to the cognitive and affective impairments induced by SCI. These results suggest that CaMKII activation may be one mechanism contributing to SCI‐induced deficits. However, the downstream pathways through which CaMKII regulates synaptic plasticity‐related transcription, as well as the upstream factors triggering CaMKII activation after SCI, remain unclear. Immunoblot results showed that phosphorylation of ERK and CREB was also elevated in the mPFC at 56 days post‐SCI. As noted above, CaMKII activation can induce ERK1/2 phosphorylation, suggesting that ERK1/2 may function as a downstream effector of CaMKII signaling and contribute to CREB phosphorylation, which has been implicated in cognitive and affective deficits following SCI. However, a definitive causal relationship among CaMKII, ERK1/2, CREB, and specific functional recovery processes has not been established. Further investigation is warranted, I suggest that. We propose that ERK1/2 activation represents a potential mechanism underlying CaMKII‐mediated effects.

SCI can dysregulate systemic immune function, which, in turn, affects the brain and alters multiple neural systems (Jaszczuk et al. 2025). SCI activates the hypothalamic–pituitary–adrenal (HPA) axis, promoting the synthesis and release of glucocorticoids (GCs) (Lerch et al. 2014). Previous studies have shown that GCs, through glucocorticoid receptor (GR)‐dependent mechanisms, modulate G‐protein‐coupled receptor (GPCR) signaling, which regulates cellular responses to biogenic amines, neurotransmitters, and peptides (Oakley et al. 2012). The diverse effects of GCs likely depend on cell type, brain region, and the timing of injury (Lerch et al. 2014). Furthermore, accumulating evidence indicates that GCs can alter glutamate neurotransmission in the mPFC, thereby influencing cognitive function (Popoli et al. 2012). GCs act via GRs, and enhanced glutamate release activates NMDARs, leading to increased intracellular Ca^2+^ levels and subsequent activation of CaMKII and the ERK1/2 MAPK cascade (Mifsud et al. 2011). Substantial evidence indicates that under conditions of SCI—which induces extensive neuroinflammation in the brain linked to cognitive and affective alterations—multiple kinase pathways, including CaMKII, become activated (Wu et al. 2014). Together, these findings and our results suggest that negative emotional behaviors and cognitive impairments following SCI might be mediated through CaMKII activation. However, further research is needed to determine how it triggers calcium activation through immune, neuroinflammatory, and synaptic plasticity changes.

In conclusion, using a well‐established rat model of SCI, we demonstrate that negative emotional behaviors and cognitive impairments are associated with elevated CaMKII activation in SCI rats. Although currently correlative, these findings suggest that CaMKII signaling may contribute to SCI‐induced cognitive decline and depression‐ or anxiety‐like behaviors. The activation of ERK1/2 and CREB may participate in this signaling pathway.

## Author Contributions

Jian Qi wrote the main manuscript text. Jian Qi prepared Figures 1, 2, 3. Chen Chen prepared Figures 4 and 5. Qian Gao prepared Figures 6 and 7. Sheng Sun prepared Figure 8. All authors reviewed the manuscript.

## Funding

This work was supported by grants from the Shandong First Medical University Teaching Program (XM2022131) and Research and Development Fund of Affiliated Hospital of Shandong Second Medical University (2024FYM106).

## Conflicts of Interest

The authors declare no conflicts of interest.

## Data Availability

The data that support the findings of this study are available from the first author, Qi Jian, upon reasonable request.
